# Phytochemical Analysis and Biological Evaluation of Carob Leaf (*Ceratonia siliqua* L.) Crude Extracts Using NMR and Mass Spectroscopic Techniques

**DOI:** 10.3390/molecules29225273

**Published:** 2024-11-07

**Authors:** Themistoklis Venianakis, Nikolaos Parisis, Atalanti Christou, Vlasios Goulas, Nikolaos Nikoloudakis, George Botsaris, Tjaša Goričan, Simona Golič Grdadolnik, Andreas G. Tzakos, Ioannis P. Gerothanassis

**Affiliations:** 1 Section of Organic Chemistry and Biochemistry, Department of Chemistry, University of Ioannina, 45110 Ioannina, Greece; vethemis@gmail.com (T.V.); nparisis@uoi.gr (N.P.); atzakos@uoi.gr (A.G.T.); igeroth@uoi.gr (I.P.G.); 2 Department of Agricultural Sciences, Biotechnology and Food Science, Cyprus University of Technology, 3603 Lemesos, Cyprus; atalanti.christou@cut.ac.cy (A.C.); n.nikoloudakis@cut.ac.cy (N.N.); george.botsaris@cut.ac.cy (G.B.); 3 Laboratory for Molecular Structural Dynamics, National Institute of Chemistry, Theory Department, Hajdrihova 19, SI-1000 Ljubljana, Slovenia; tjasa.gorican@ki.si (T.G.); simona.grdadolnik@ki.si (S.G.G.)

**Keywords:** antimicrobial activity, carbohydrate digestive enzyme inhibition, carob leaf, flavonoids, galloyl derivatives, myricetin, mass spectrometry, NMR spectroscopy

## Abstract

Carob leaves have gained attention for their bioactive properties and traditional medicinal uses, including as treatment for diabetes, digestive disorders, and microbial infections. The aim of this study was to explore the phytochemical composition of carob leaf acetone extracts using advanced spectroscopic techniques. The combined use of heteronuclear nuclear magnetic resonance (NMR) experiments with 1D selective nuclear Overhauser effect spectroscopy (NOESY) offers detailed structural insights and enables the direct identification and quantification of key bioactive constituents in carob leaf extract. In particular, the NMR and mass spectrometry techniques revealed the presence of myricitrin as a predominant flavonoid, as well as a variety of glycosylated derivatives of myricetin and quercetin, in acetone extract. Furthermore, siliquapyranone and related gallotannins are essential constituents of the extract. The potent inhibitory effects of the carob leaf extract on *Staphylococcus aureus* (MIC = 50 μg mL^−1^) and a-glucosidase enzyme (IC_50_ = 67.5 ± 2.4 μg mL^−1^) were also evaluated. Finally, the antibacterial potency of carob leaf constituents were calculated in silico; digalloyl-parasorboside and gallic acid 4-*O*-glucoside exert a stronger bactericidal activity than the well-known myricitrin and related flavonoids. In summary, our findings provide valuable insights into the bioactive composition and health-promoting properties of carob leaves and highlight their potential for pharmaceutical and nutraceutical applications.

## 1. Introduction

The carob tree (*Ceratonia siliqua* L.) is one of the most representative plants of agro-forest ecosystems in the Mediterranean basin, where it is considered an important component of the area’s vegetation for environmental and economic reasons [1]. It is an evergreen xerophyte tree that is native to the arid and semi-arid Mediterranean region, contributing to the sustainability of Mediterranean ecosystems [1]. The carob tree produces large pods which are rich in sugars, fibers, and bioactive compounds like cyclitols and polyphenols. Special attention is also given to carob seeds due to their content of carob (locust) bean gum, which has multiple applications in the food and pharmaceutical sectors [2]. Apart from studies focused on the edible parts of carob fruit, other studies have considered the constituents present in carob leaf extracts, mainly because of their availability and low raw material cost in Mediterranean countries. In integrative medicine, carob leaves are traditionally used for the treatment of diabetes, digestive diseases, gastrointestinal disorders, and herpes [3]. Furthermore, previous studies highlight the potent inhibitory effect of carob leaf extracts against tumor cell growth, enzymes related to diabetes and inflammation, and bacteria growth, as well as their protective effects against inflammation, colorectal cancer, and oxidative stress [4,5,6,7,8]. The biological and health effects of carob leaf extracts are mostly correlated with the presence of a variety of phenolic compounds contained in the extracts. Hydroxycinnamic and hydroxybenzoic acids, as well as flavonoids, are found in substantial concentrations in carob leaves, although they vary greatly, being influenced by genetic and environment factors [3,5,7,9].

Flavonoids are a fascinating group of phenolic compounds due to their exceptional bioactivity and structural diversity. In hydrophilic extracts of carob leaves, many derivatives of quercetin, naringenin, apigenin, kaempferol, catechin, and myricetin were mainly identified. Previous works have highlighted the presence of a glycosylated derivative of myricetin, known as myricitrin, in carob leaves [3,5,7,9]. Myricetin-3-*O-*α-rhamnopyranoside (myricitrin) is a naturally occurring flavonoid glycoside belonging to the flavonol subgroup. It exerts multiple physiological activities, such as antioxidant, anti-inflammatory, anti-cancer, anti-diabetic, and cardio-/neuro-/hepatoprotective activities, due to the five hydroxyl groups on its structure [10]. The identification and quantification of myricitrin and its derivatives in carob leaf extracts was previously performed with the employment of chromatographic techniques coupled with UV–vis and/or mass spectrometric detection, although the chromatographic separation was complicated [5,11]. Phenolic acids are also widely distributed in carob leaves; gallic acid and its glycosylated derivatives have been identified [11,12] Gallotannins are also typical phytoconstituents in carob leaves, and siliquapyranone, a tannic acid tetrahydropyran-2-one, was recently isolated from aqueous carob leaf extract as well [13,14].

Among the spectroscopic methods commonly used in the analysis of natural products, NMR spectroscopy is a dominant technique due to the wide range of experimental approaches that are available, the high level of structural information that is provided, and the non-destruction of the sample [15,16]. Significant effort has been given to the development of NMR methodologies that can be applied successfully to the analysis of various phytoconstituents of crude plant extracts without any previous separation or isolation of the individual components [17,18]. The present work aims to couple state-of-the-art NMR methodologies with mass spectrometry experiments for the detailed phytochemical analysis of carob leaf acetone extract (CLAE). Furthermore, the biological potency of the extracts in terms of the antibacterial and digestive enzyme inhibition effects of the extract were assessed.

## 2. Results and Discussion

### 2.1. DNA Barcoding of Ceratonia siliqua L. Leaves

The germplasm was first characterized based on previously reported keys and morphological descriptors [19]. The leaves were pinnately placed alongside the main vein, having no terminal leaflet. The leaflets (3–7 cm long) were ovate to elliptic, ranging from 4 to rarely 10 cm. The adaxial surface was leathery, dark green, and shiny, while the abaxial side had a pale green hue. The rRNA/ITS (PQ303363) sequenced fragment was 731 bp, containing the small subunit ribosomal RNA, the internal transcribed spacer 1, the 5.8S ribosomal RNA, and the internal transcribed spacer 2. We conducted a Blastn query, which had a 99.39% identity match to the previously published *Ceratonia siliqua* L. accession MT113332.1, and an e-value of 0.0; thus, the sample’s morphological identification as a carob tree was fully supported. The second barcoding marker (PQ308987) was bp 711 long, and a 99.72% identity match to the *Ceratonia siliqua* L. voucher Yi15508-KUN chloroplastic genome was established after querying the nr database (e-value of 0.0). The concurrent analyses indicated significant alignments, thus suggesting a phylogenetic identity.

### 2.2. NMR and MS Study of Carob Leaf Acetone Extract

**Figure 1:**
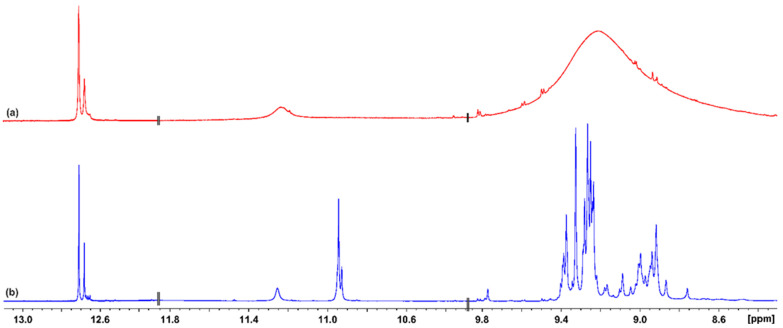
(**a**) 500 MHz ^1^H NMR spectrum of the phenol-OH region of 20.14 mg carob leaf acetone extract in 500 μL DMSO-d_6_. (**b**) The same spectrum as in (**a**) after titration with trifluoroacetic acid solution in DMSO-d_6_. NMR spectra were acquired at 295 K and with 64 scans, 1.9 s acquisition time, and a relaxation delay of 4.0 s.

**Figure 2:**
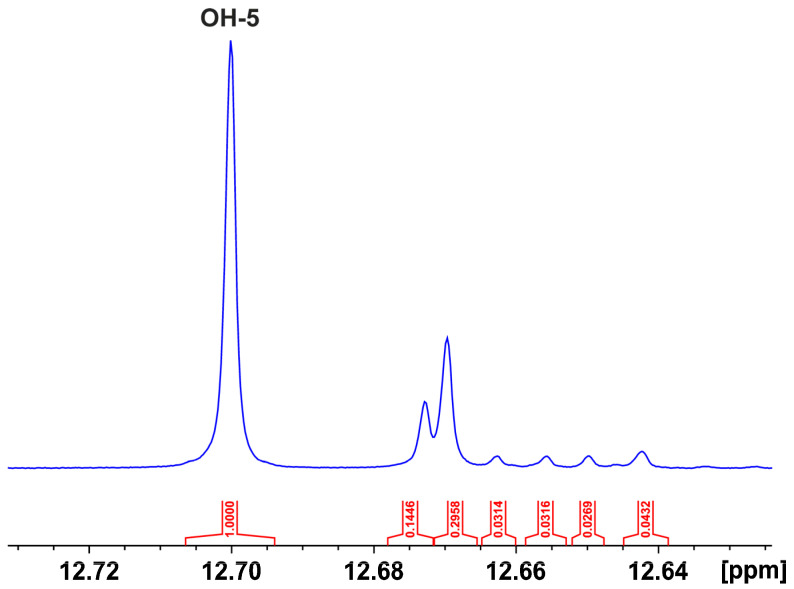
800 MHz ^1^H NMR spectrum of the expanded OH(5) region of flavonoids and their integrals relative to that of the major analyte myricetin-3-*O*-α- rhamnopyranoside (12.700 ppm).

**Figure 3:**
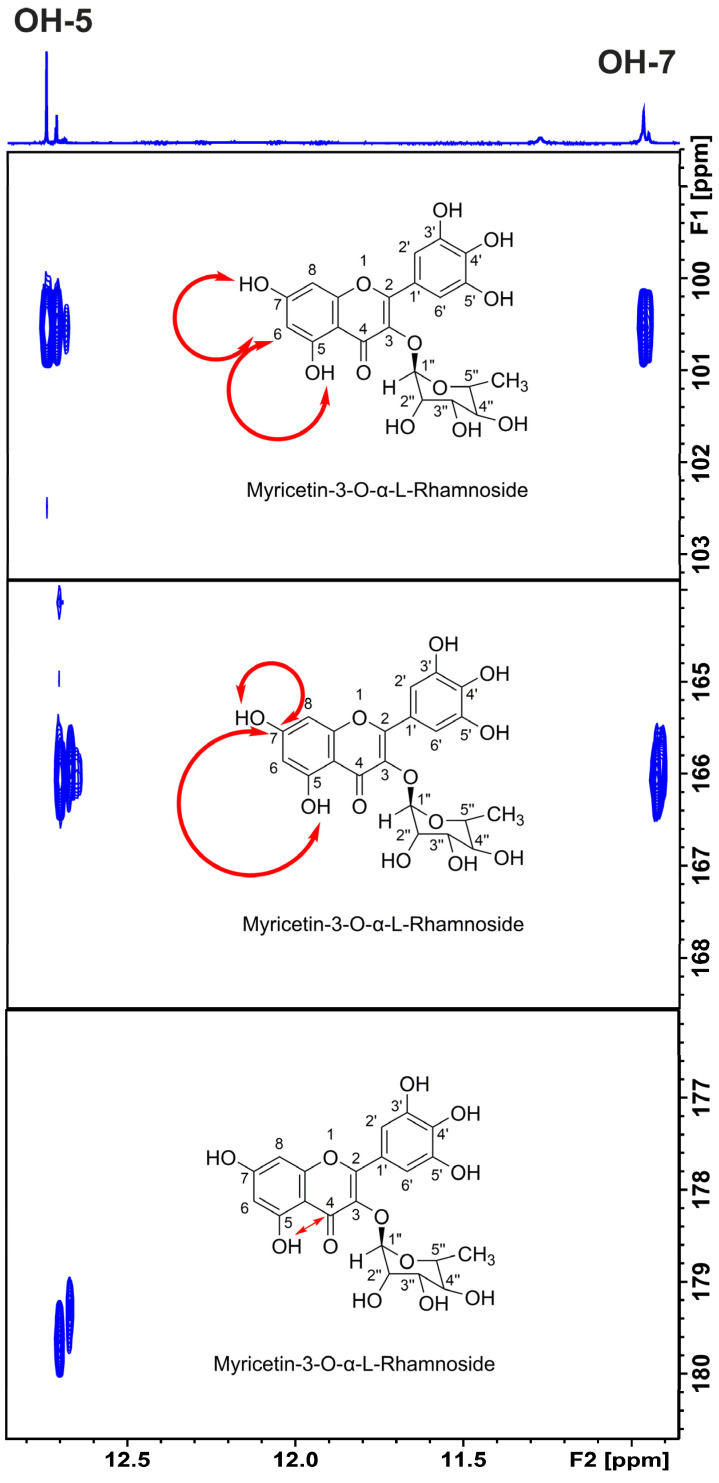
800 MHz ^1^H-^13^C HMBC spectrum of the selected region of 20.14 mg carob leaf acetone extract in 500 μL DMSO-d_6_. Selected region of 800 MHz ^1^H-^13^C HMBC spectrum of 20.14 mg carob leaf acetone extract in 500 μL DMSO-d_6_. The double arrows denote the connectivities of -OH(5) and -OH(7) with characteristic carbon atoms of myricetin-3-*O*-α-l-rhamnopyranoside. The spectrum was acquired at 298 K and with 56 scans and 1024 increments. The total experimental time was 14 h and 30 min.

The 1D ^1^H NMR spectrum of the CLAE in acidified DMSO-d_6_ with trifluoroacetic acid indicates a significantly enhanced resolution of the phenοl -OH groups compared to those in ordinary DMSO-d_6_ (Figure 1). The use of the 800 MHz instrument resulted in several very sharp resonances (Δν_1/2_ < 2.0 Hz) with excellent resolution in the strongly deshielded region above 11 ppm (Figure 2), which can be attributed to OH(5) protons of flavonoids, which participate in a strong intramolecular hydrogen bond with the oxygen atom of the carbonyl CO(4) group [20]. Three major flavonoids were detected, with chemical shifts of 12.700, 12.673, and 12.670 ppm and relative integrals of 1.00:0.14:0.30, respectively. The resonances at 12.663, 12.656, 12.650, and 12.642 ppm have relative integrals <0.04 compared to that of the major analyte at 2.700 ppm (Figure 2). The sharp -OH resonances allowed the application of the ^1^H–^13^C heteronuclear multiple bond correlation (HMBC) experiment. The OH(5) proton of the major analyte at 12.70 ppm shows six cross peaks at C_5_ (163.21 ppm), C_6_ (100.54 ppm), C_7_ (166.06 ppm), C_9_ (158.34 ppm), C_10_ (105.89 ppm), and C_4_ (179.61 ppm) (Figure 3). The OH(7) at 10.94 ppm shows a smaller number of cross peaks at C_5_ (163.21 ppm), C_6_ (100.54 ppm), C_7_ (166.06 ppm), C_8_ (95.38 ppm), and C_9_ (158.34 ppm) due to having a larger line width (Δν_1/2_ ~ 3.7 Hz). The common ^13^C cross peaks of the OH(5) and OH(7) protons demonstrate that they belong to the same ring system (Figure 3). The cross peak of the OH(5) at CO(4) is, also, of high diagnostic value due to the great sensitivity of δ(^13^C) to conjugation. Thus, the strong shielding at 179.61 ppm indicates the presence of a C_2_–C_3_ double bond; the absence of the double bond resulted in δ(^13^C) in the range of 196–199 ppm [20].

**Figure 4:**
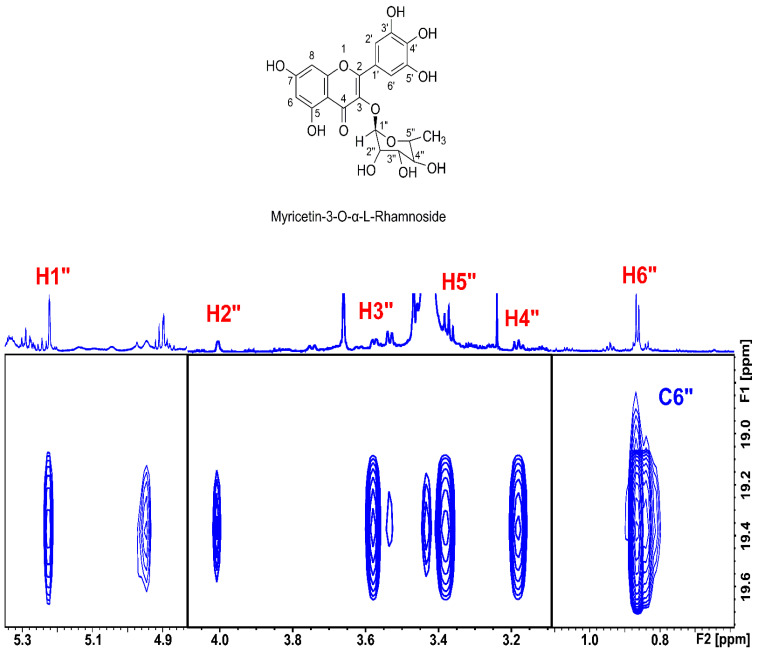
800 MHz HSQC-TOCSY spectrum of selected region of 20.14 mg carob leaf acetone extract in 500 μL DMSO-d_6_. Characteristic connectivities between the methyl carbon and all protons of the α-rhamnopyranoside ring are shown. The spectrum was acquired at 298 K and with 64 scans, 1024 increments, and a mixing time of 100 ms. The total experimental time was 1 day and 7 h.

The assignment of the glycoside moiety of the major analyte was based on the characteristic chemical shifts of the C_5″_–CH_3_ group (δ(^1^H) = 0.87 ppm, δ(^13^C) = 19.38 ppm). Application of the 2D heteronuclear single quantum coherence-total correlation spectroscopy (HSQC-TOCSY) experiment (Figure 4) allowed the complete identification of the rhamnopyranoside moiety (Table 1). The anomeric configuration of hexopyranoses can be deduced from the magnitude of the ^3^J(H_1″_, H_2″_) coupling constants [21]. A large ^3^J(H_1″_, H_2″_) in the range of 7.8–8.5 Hz indicates a β-configuration, whereas ^3^J(H_1″_, H_2″_) < 4Hz implies an α-configuration. This distinction, however, is not possible for pyranosyl rings where the H_2″_ is oriented equatorially, such as in methyl glycosides, where both α- and β-anomeric configurations result in small (<2 Hz) ^3^J(H_1″_, H_2″_) coupling constants. Figure 4 shows the value for the ^3^J(H_1″_, H_2″_) coupling constant ~1.70 Hz. In this case, the configuration of the anomeric carbons can be deduced unequivocally from the ^1^J (C_1″_, H_1″_) coupling constants (~170 Hz and 160 Hz for α- and β-configuration, respectively) [22]. The ^1^H-^13^C HSQC experiment showed a ^1^J(C_1″_, H_1″_) coupling constant of ~172.3 Hz and, thus, the α-configuration. The ^1^H NMR chemical shift data shown in Table 1 are in excellent agreement with the data found for isolated myricetin-3-*O*-α-l-rhamnopyranoside by Motlhatlego et al. [23] and Castro et al. [24], but at variance with those of Blunder et al. [25] and Wu et al. [26].

**Figure 5:**
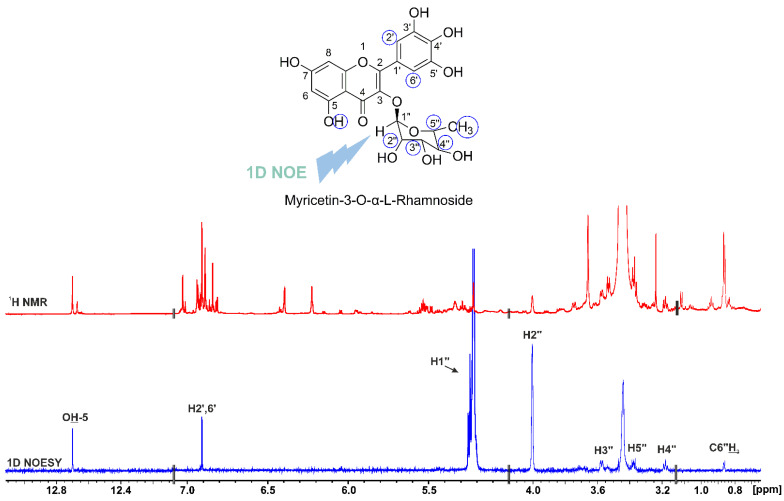
800 MHz 1D selective NOESY NMR spectrum of 20.14 mg carob leaf acetone extract in 500 μL DMSO-d_6_. The irradiated proton of myricetin-3-*O*-α-l-rhamnopyranoside is denoted with thunder and the protons showing connectivities are circled. The spectrum was acquired at 298 K and with 256 scans and a mixing time of 600 ms. The total experimental time was 30 min.

Unfortunately, ^1^H-^13^C HMBC correlation was not observed between H_1″_ and C_3_, C_4_, and C_2_; thus, our identification of the glycoside site was based on the 1D selective nuclear Overhauser effect spectroscopy (NOESY). Selective excitation of the H_1″_ results in NOE connectivities between all protons of the rhamnopyranoside moiety (most notably that of H_2″_) and the H_2′,6′_ of ring B, and the OH(5) of ring A (Figure 5). Therefore, this 1D NOESY experiment could be of general interest for use in investigating the site of glycosylation in flavonoids due to its simplicity and significant time-saving potential. Figure 5 shows that NOE signals with a very good signal to noise ratio can be achieved within 30 min.

In addition, the high-resolution ^1^H-NMR spectroscopy of the –OH spectral region was utilized as a rapid method for the direct quantification of the major phytoconstituents in CLAE without any prior isolation step. It was based on the integration of the OH-5 signal relative to the resonance of the reference compound 3-(trimethylsilyl)propionic-2,2,3,3-d_4_ acid sodium salt, which was of known concentration. The concentration of the major analyte myricetin-3-*O*-α-l-rhamnopyranoside was 179 mg g^−1^ (17.9% *w*/*w*) of dry extract. The present extract is richer in myricetin-3-*O*-α-l-rhamnopyranoside than polar extracts (1.7–38 mg g^−1^), as flavonoids possess a solubility preference to acetone compared to alcohols [5,11].

The second major flavonoid at 12.670 ppm has an integral of ~30% compared to that of myricetin-3-*O*-α-l-rhamnopyranoside (δ = 12.700 ppm, Figure 2). The ^1^H-^13^C HMBC spectrum (Figure S1) shows identical cross peaks of the OH(5) with C7 and C5. In addition, the CO(4) connectivity (δ = 179.34 ppm) shows the presence of a C2–C3 double bond, and the shielding by 0.27 ppm relative to that of myricetin-3-*O*-α-l-rhamnopyranoside also indicates the presence of a myricetin derivative with a different glycoside moiety. This analyte was shown by MS to be myricetin-3-*O*-galactopyranosides (see below). This was further confirmed by the ^1^H-^13^C HSQC and HMBC connectivity of the anomeric CH1″ group (δ(^13^C) = 103.69 ppm and δ(^1^H) = 5.27 ppm (Figure 4)) [27,28].

Of particular interest is the ratio of the total integral of the OH(5) resonances relative to the total integral of the phenol OH resonances in the region of 8.6–9.6 ppm (the contribution of myricetin derivatives was not taken into account), which was found to be 1.0/10.0. This also demonstrates the significant contribution of phenolic, non-flavonoid compounds (see discussion below).

The phytochemical composition of the extract was also determined using an ultra-performance liquid chromatography system coupled with a photodiode array detector and an electrospray ionization quadrupole time-of-flight mass spectrometry system (UPLC-PDA-ESI/QToF), expanding our previously published methodology [29]. Figure S2 shows total ion account (TIC) mass chromatograms at 254 nm, 280 nm, and 360 nm (flavonoids). An extensive review of the existing literature on the phytochemical composition of *Ceratonia siliqua* L. was carried out, leading to the creation of a compound structure database from previously identified compounds [13,30,31,32]. Three major flavonoid compounds were identified in the extract, with myricitrin (myricetin-3-*O*-α-l-rhamnopyranoside) being the predominant flavonoid compound (Figure S2), in excellent agreement with the NMR results. The second major flavonoid was myricetin-3-*O*-galactopyranoside; its content was 5.6% *w*/*w* as estimated by the NMR experiments. Both compounds exhibited the characteristic myricetin fragment at *m*/*z* 316. Additionally, quercitrin (quercetin-3-*O*-α-l-rhamnopyranoside) was identified, showing the typical quercetin fragment at *m*/*z* 300. All three flavonoids were confirmed through retention times and mass spectra comparison with reference standards. Furthermore, myricetin-pentoside and a kaempferol/luteolin-deohexoside were detected based on fragment ions at *m*/*z* 316 and *m*/*z* 285, respectively. These compounds were confirmed as flavonoids by their UV spectra, which displayed characteristic absorption bands at 347–356 nm and 254–265 nm. These findings are consistent with previous reports in the literature [30,32].

In addition to the flavonoids, nine compounds were identified as gallic acid derivatives, primarily gallotannins, which were characterized by their fragment ion at *m*/*z* 169, corresponding to the gallic acid moiety (Figure S3 and Table 2). The predominant gallotannin was identified as digalloyl-parasorboside (also reported as siliquapyranone), with prominent fragment ions at *m*/*z* 169, 595, 443, and 125 [13]. The presence of siliquapyranone was also confirmed by NMR, showing the characteristic chemical shift of the methyl group at 1.33 ppm (d, J 6.35 Hz), the ^1^H–^13^C HMBC correlations of the ester carbonyl resonances at 70.80 ppm, and the methylene resonance at 2.66 ppm (dd, J 16.0 and 4.9 Hz) of the 5-hexanolide ring (Figure S3). Furthermore, the ^1^H–^13^C HMBC correlations between the methine ^1^H resonances of the β-glucopyranosyl ring at 5.33 ppm (J 8.1 Hz) and the ester carbon resonance at 166.51 ppm and between the ^1^H resonance at 5.29 ppm (J 9.1 Hz) and the ester carbon resonance at 166. 91 ppm supported the presence of two galloyl moities [13]. Additional evidence was also provided by the HMBC correlations of the singlet aromatic protons at 6.84 ppm and 6.89 ppm with the ester carbon resonances. Siliquapyranone is the major gallotanin in CLAE, as its content was determined at 13.7% *w*/*w*. Galloyl-parasorboside was also detected, characterized by fragments at *m*/*z* 169, 331, and 313. Additionally, two digalloyl-glucose isomers were identified by fragments at *m*/*z* 169, 331, and 313, three trigalloyl-glucose isomers were identified by fragments at *m*/*z* 169, 465, and 313, and two tetragalloyl-glucose isomers were identified by fragments at *m*/*z* 169, 617, 465, and 313. Among the major compounds, epigallocatechin 3-*O*-gallate was also identified, showing fragments at *m*/*z* 169, 125, and 305, as well as gallic acid itself, which displayed fragments at *m*/*z* 125 and 151. All identified gallic acid derivatives have been previously described in the literature [13,30,31,32], except for galloyl-parasorboside, for which, in contrast to siliquapyranone (digalloyl-parasorboside), its presence in *Ceratonia siliqua* L. has never been described before.

### 2.3. Biological Evaluation of Carob Leaf Acetone Extract

At first, the inhibitory effects of CLAE acetone extract on carbohydrate digestive enzymes, namely alpha-glucosidase and alpha-amylase, were assessed. Both enzyme inhibitions are considered as well-established biomarkers of possible antidiabetic effects. Alpha-glucosidase is able to catalyze the cleavage of disaccharides to glucose. The inhibitors of this enzyme can retard the uptake of dietary carbohydrates and suppress postprandial hyperglycemia. In addition, alpha-amylase is responsible for transforming dietary starch to glucose prior to absorption. Its inhibition can lead to a reduction in post-prandial hyperglycemia in diabetes. Therefore, the inhibition of both enzymes is considered an effective strategy to control diabetes [33]. CLAE was more effective in inhibiting the activity of alpha-glucosidase than alpha-amylase, since lower concentrations of the extract are required. In particular, the IC_50_ (half-maximal inhibitory concentration) of the extract was 67.5 ± 2.4 μg mL^−1^ against alpha-glucosidase. Custódio et al., in 2015, reported the in vitro inhibitory activity of water decoctions of carob on α-glucosidase [6], but the potential of acetonic extract is demonstrated for first time herein. The results clearly demonstrated that the inhibitory effect of the extract was less efficient against alpha-amylase than alpha-glucosidase, although a higher concentration of extract was used. More specifically, the IC_50_ of the extract is higher than 500 μg mL^−1^; thus, it cannot be considered as a promising inhibitor of amylase enzyme. The partial absorption of the extract’s constituents by starch during gelatinization, and different interactions between the enzyme and active constituents, may explain the different responses of the digestive enzymes [34]. According to the literature, myricetin-3-*O*-α-rhamnopyranoside and other glycosidic derivatives demonstrated potent inhibitory effects on alpha-glucosidase and alpha-amylase, highlighting their significant contributions to the potential of CLAE extract to act as an inhibitor of both enzymes [35,36]. Regarding the predominant gallotannin, namely digalloyl-parasorboside, its inhibitory effects on carbohydrate digestive enzymes is unknown yet. However, trigalloylglucose and tetragalloylglucose isomers have been evaluated as potent alpha-glucosidase inhibitors [37,38].

The inhibitory effects of CLAE against a panel of important foodborne bacteria were also investigated. The results showed a weak antimicrobial potency against Gram-negative bacteria, namely *Cronobacter sakazakii*, *Escherichia coli*, and *Salmonella enterica*, since minimum inhibitory concentration (MIC) values higher than 2000 μg mL^−1^ were found (preliminary data). On the other hand, Gram-positive bacteria (*Bacillus cereus*, *Listeria monocytogenes*, and *Staphylococcus aureus*) were susceptible to CLAE at lower concentrations (Table 3). Among the Gram-positive bacteria tested, the potent inhibitory effect against *Staphylococcus aureus* was distinguished. More specifically, the MIC and minimum bactericidal concentration (MBC) values of the CLAE were 50 and 150 μg mL^−1^, respectively. Both the MIC and MBC values highlighted the great potential of the extract to act as a growth inhibitor of *S. aureus*, since they meet stringent end point criteria for the “activity” of plant extracts, as suggested by Cos and co-authors (2006) [39]. The antibacterial potency of carob leaves has been evaluated against several bacteria; however, only one attempt has been made to assess the potential of carob leaves extracts against *Staphylococcus* strains. More specifically, ethanol carob leaf extract significantly inhibited the growth of *S. aureus* ATCC 6538, with inhibition zones comparable to those of amoxicillin and imipenem [9]. Regarding the MIC and MBC values, the potential of the CLAE was remarkably higher for the same strain of *Staphylococcus*. Furthermore, the inhibitory effect of carob leaves on *S. aureus* is stronger than that of carob fruits [40]. Its strong antibacterial potency is correlated with the major phytoconstituent, namely myricetin-3-*O*-α-rhamnopyranoside, as it is a substantial growth inhibitor of *Staphylococcus* bacteria. Previous studies reported MIC values that ranged from 62.5 to 125 μg mL^−1^ [23,41]. Considering the MIC of myricetin-3-*O*-α-rhamnopyranoside, there are other antimicrobial compounds in the extract that could act with additive or synergistic effects. Thus, the antibacterial activities of major constituents of the extract against *S. aureus* were calculated using the AntiBac-Pred web tool of the Way2Drug platform. Antibac-Pred analyses the growth inhibitors or non-inhibitors of *S. aureus* based on antibacterial activity data available in ChEMBL (a database of bioactive molecules with drug-like properties). The score for each phytoconstituent is expressed as confidence in its activity, which is the difference between the probabilities that a chemical compound inhibits or does not inhibit the growth of a given bacteria. As confidence increases, the chances of the prediction being true are greater [42]. The computational screening demonstrated that digalloyl-parasorboside and gallic acid 4-*O*-glucoside exert stronger bactericidal activity than myricitrin and related flavonoids (Table 4) [43]. Furthermore, the antibacterial potency of carob leaf gallotannins is comparable with that of myricetin derivatives, which are well-known antimicrobial agents. Although the inhibitory effects of gallotannins have been studied, the antibacterial potency of digalloyl-parasorboside has not been investigated yet.

## 3. Materials and Methods

### 3.1. Plant Collection and Characterization

Leaves of *Ceratonia siliqua* L. were harvested from Limassol District, Cyprus (34°41′50.2″ N 32°57′40.5″ E) on 1 July 2022. Voucher specimen was prepared and is housed in the department’s herbarium. For the barcoding documentation, a two-locus approach was followed as previously advised [44]. The internal transcribed spacers were chosen as the nuclear (ITS) and the large subunit of ribulose bisphosphate carboxylase (rbcL) was chosen as a chloroplastic marker, due to their proven discriminating capacity across land plants [45]. To amplify the nuclear rDNA locus via polymerase chain reaction (PCR), the universal plant-specific primers 5′-TAG AAT TCC CCG GTT CGC TCG CCG TTA C-3′ (26S) and 5′-ACG AAT TCA TGG TCC GGT GAA GTG TTC G-3′ (18S) were employed. The chloroplastic locus was amplified using the common rbcl primers: 5′-ATG TCA CCA CAA ACA GAA AC-3′ (rbcL1F) and 5′-TCG CAT GTA CCT GCA GTA GC-3′ (rbcL724R). For the PCR setup, each reaction (50 µL) contained 10 ng of genomic DNA as a template, 10 pmol of each forward/reverse primer, and 1X KAPA HiFi HotStart ReadyMix (Roche Molecular Systems, Pleasanton, CA, USA) which featured a high-fidelity polymerase. The cycling profile was based on the manufacturer’s recommendation and composed of an initial denaturation step at 95 °C for three min, followed by 35 cycles (30 s at 95 °C, 30 s at 55 °C, and one min at 72 °C). A final elongation step for 5 min (at 72 °C) was also included before cooling to 8 °C. The resulting amplicons were evaluated by standard 1% agarose gel electrophoresis, and both strands were sequenced (Eurofins, Luxembourg, Luxembourg). Resulting sequences were curated to assemble one strand with a plus/plus configuration. Similarities to deposited taxa were investigated using the Basic Local Alignment Search Tool (NCBI) and the Blastn algorithm on the core nucleotide database (assessed in September 2024).

### 3.2. Plant Extraction

*Ceratonia siliqua* L. leaves were dried in a conventional oven at 40 °C until constant weight. Finally, dried leaves were pulverized with the employment of an electric grinder, Sage BCG820BSSUK (Breville Group Limited, Sydney, Australia). An amount of 5 g of dry powdered leaves was firstly mixed with 30 mL of hexane. The mixture was placed in an ultrasonic bath and sonicated for 60 min at 60 °C. After the ultrasound treatment, the mixture was allowed to cool at room temperature and then centrifuged for 10 min at 2500 rpm. The supernatant was discarded and the remaining solid was extracted again with 30 mL of acetone. The mixture was placed again in an ultrasonic bath and sonicated for 60 min at 40 °C. Subsequently, the mixture was allowed to cool and then centrifuged for 10 min at 2500 rpm. The supernatant was collected, and the solvent of the extract was removed using nitrogen gas.

### 3.3. NMR Experiments

NMR experiments were performed at 298 K on a Bruker Avance Neo 500 spectrometer (University of Ioannina) equipped with a broadband inverse probe (Bruker Biospin, Rheinstetten, Germany) and a Bruker Avance Neo 800 spectrometer equipped with a cryogenically cooled probe (Slovenian NMR Centre, National Institute of Chemistry, Ljubljana, Slovenia). Samples were dissolved in 0.6 mL of DMSO-d6 and transferred to 5 mm NMR tubes. The NMR spectrometer was controlled by the software TopSpin 4.0.9 (Bruker BioSpin, Rheinstetten, Germany). The 2D ^1^H–^13^C HSQC and HMBC and the 2D HSQC-TOCSY experiments were carried out using the above software. Parameters were optimized for coupling constants of 145.0 Hz and 10.0 to 2.5 Hz, respectively. TOCSY mixing time of 100 ms was used. One-dimensional selective NOESY and TOCSY experiments were recorded at 298 K on a Bruker Avance Neo 800 MHz spectrometer with 600 ms and 100 ms mixing times, respectively.

### 3.4. Chromatography (Photodiode Array/Mass Spectrometry) Experiments

The extract underwent appropriate dilution and filtration with a 0.2 μm RC filter prior to its introduction to the chromatography system. The separation of phytochemical compounds was conducted using an ACQUITY iClass Plus UPLC system (Waters, Manchester, UK), which included a temperature-controlled autosampler set at 6 °C and an Acquity T3-HSS C18 column (100 mm × 2.1 mm i.d., 1.7 μm particle size) maintained at 45 °C. The mobile phase was a mixture of 0.1% formic acid in water (solvent A) and acetonitrile with 0.1% formic acid (solvent B), delivered at a flow rate of 0.4 mL/min. The gradient elution schedule was set as follows: from 0.00 to 0.07 min, 1% B; from 0.07 to 10.00 min, a linear increase from 1% to 100% B; held at 100% B from 10.00 to 12.67 min; returned to 1% B from 12.67 to 12.73 min; and re-equilibrated at 1% B from 12.73 to 15.00 min. The volume of the injected sample was 2 μL. An ACQUITY Premier Photodiode Array (PDA) eLambda detector (Waters, Manchester, UK) was connected to the UPLC system. A full spectrum acquisition was performed in a range from 220 to 600nm with a scan time of 0.2s. Individual chromatographs were also recorded at the wavelengths of 254, 280, and 360 nm. The Xevo G2-X2 Q-ToF mass spectrometer (Waters, Manchester, UK) was connected directly to the output of the PDA system via an ESI interface, and was operated in negative ion mode. Optimal ionization conditions were set as follows: capillary voltage at 1.0 kV, sample cone voltage at 40 V, source temperature at 120 °C, desolvation temperature at 550 °C, cone gas flow at 20 L/h, and desolvation gas flow at 1000 L/h. The LockSpray feature was used for consistent and precise measurements, with leucine-enkephalin serving as the lockmass at a concentration of 5 ng/mL, producing a reference ion at *m*/*z* 554.2620 that was infused at 10 μL/min. Data collection was performed in MS^E^ mode, with two separate scanning functions: Function 1 (low energy), with a mass range of 50–1200, a scan time of 0.2 s, and a collision energy of 4 eV; and Function 2 (high energy), with the same mass range and scan time and a collision energy ramp from 25 to 45 eV. This dual-function scanning provided simultaneous data on intact precursor ions and their fragments. The data acquisition on the UPLC-PDA-QToF system was controlled by MassLynx 4.2 software (version SCN 1018) and data processing was carried out using the UNIFI software platform (version 1.9.4.053). Compound identification was carried out by comparing the UV absorption data from the PDA detector, the mass spectrometry data and fragmentation patterns from the ESI/QToF mass spectrometer, and the retention times from both reference standards and a proprietary compound library.

### 3.5. Antibacterial Properties

Three Gram-positive bacteria, namely *Listeria monocytogenes* ATCC 23074 (serotype 4b), *Staphylococcus aureus* ATCC 6538, and *Bacillus cereus* ATCC 6089, were grown in Listeria Agar (Merck^®^, Darmstadt, Germany), Baird Parker Agar (Liofichem^®^, Roseto degli Abruzzi, Italy), and Mannitol Egg Yolk Polymyxin (MYP) Agar (Merck^®^, Darmstadt, Germany), respectively. Furthermore, three Gram-negative bacteria, namely *Salmonella enterica* subsp. *enterica* serovar Enteritidis NCTC 5188, *Escherichia coli* ATCC 11775, and *Cronobacter sakazakii* ATCC 29544, were grown in Xylose Lysine Deoxycholate Agar (Merck^®^, Darmstadt, Germany), Tryptone Bile Glucuronic Agar (Merck^®^, Darmstadt, Germany), and Sakazakii Agar (Merck^®^, Germany), respectively. A colony of each of the bacteria was inoculated into 10 mL Brain Heart Infusion Broth (BHI) (Himedia^®^, Mumbai, India) and incubated at 37 °C. Antibacterial susceptibility test was performed using the broth microdilution method with slight modifications. More specifically, an aliquot of 50 μL of extract was transferred, in triplicate, to a 96-well plate. An aliquot of 40 μL of BHI and 10 μL of microbial suspension were added to reach a final volume of 100 μL in each well. Microbial suspensions were adjusted so that the final concentration in the wells was 10^6^ cfu mL^−1^. Screening for bacteriostatic/bactericidal activity of plant extracts was performed as described in previous works by adding 10 μL from each well into BHI agar plates, and the results were extracted after incubation for 24 h at 37 °C [46]. Controls of 10%, 5%, and 2.5% *v*/*v* DMSO and microbial cultures were also tested. Stock solutions of 10 mg mL^−1^ for extract were prepared using DMSO as a diluent. Stock solutions were further diluted using water to prepare working solutions. Bacteriostatic activity was expressed as minimum inhibitory concentration (MIC) and bactericidal activity as minimum bactericidal concentration (MBC). MIC and MBC values were calculated as μg dry extract mL^−1^.

### 3.6. Inhibitory Potential on Diabetes-Related Enzymes

The determination of the alpha-glucosidase inhibitory effect of plant extracts was assessed by mixing the diluted extract solution (100 μL) with 50 μL of 0.1 mM phosphate buffer (pH = 6.8) containing alpha-glucosidase (1.0 U mL^−1^). The mixture was incubated at 37 °C for 10 min. Subsequently, 50 μL of *p*-nitrophenyl-α-d-glucopyranoside (PNG) (5 mM in 0.1 mM phosphate buffer, pH = 6.8) was added and the reaction mixture was allowed to stand for 5 min. Finally, the absorbance was measured at 405 nm against a blank solution where PNG was replaced with buffer. Control, which represented 100% enzyme activity, was prepared by replacing the extract solution with 20% (*v*/*v*) DMSO.

For the assessment of alpha-amylase inhibitory potential, 100 μL of the diluted extract solution and 100 μL of alpha-amylase solution (2 U mL^−1^ in 20 mM sodium phosphate containing 6.7 mM NaCl, pH 6.9) was incubated at 35 °C for 10 min. Then, 200 μL of soluble starch (1% *w*/*v* in the same buffer) was added and the mixture was incubated again at 35 °C for 20 min. The reaction was terminated by adding 200 µL of 3,5-dinitrosalicylic acid (DNS) reagent. Afterwards, the mixture was boiled for 10 min, cooled down, and diluted with deionized water (1:10, *v*/*v*) before the measurement of absorbance at 540 nm. The absorbance of the reaction mixture was measured against a blank sample containing the extract solution, starch solution, and DNS (without enzyme). Results for both assays were expressed as the half-maximal inhibitory concentration (IC_50_) [46].

### 3.7. In Silico Screening Antimicrobial Potency of Phytoconstituents

The inhibitory effects of CLAE constituents against *S. aureus* were also calculated. The two-dimensional structures and canonical SMILES of the compounds were obtained from the PubChem Compound page at https://pubchem.ncbi.nlm.nih.gov/, accessed on 10 September 2024. The antibacterial activities against the *S. aureus* were determined using the AntiBac-Pred web tool of the Way2Drug platform (https://www.way2drug.com/antibac/), accessed on 10 September 2024 [47].

## 4. Conclusions

The present work demonstrates a comprehensive strategy for the detailed phytochemical analysis of CLAE with the employment of the state of the art of NMR and MS experiments. The proposed strategy allows the direct identification and quantification of a variety of flavonols and gallotannins without the need for laborious isolation and purification procedures for the individual phytoconstituents. This combined use of heteronuclear NMR experiments with 1D NOESY experiments and mass spectrometry measurements can be easily applied for the screening of the phytochemical compositions of carob leaves of different cultivars and genders, or to screen carob leaves grown under different environmental conditions. Furthermore, it is an extremely useful tool for the development of a prototype for the standardization of carob leaf extract. In addition, the evaluation of the bacteriostatic and bactericidal potency of CLAE against a panel of bacteria showed that it can be used to efficiently control the growth of the *Staphylococcus aureus* bacterium. This strong inhibitory effect of CLAE is attributed to the presence of gallic acid derivatives as well as myricitrin, as revealed by in silico measurements. The findings highlight that these phytochemicals could be used as lead compounds toward the discovery of new bactericidal agents for *Staphylococcus aures*. Finally, CLAE can act as a strong alpha glucosidase inhibitor and can thus be used for the management of diabetes mellitus. The fractionation and purification of CLAE may be a possible route to improving its inhibitory effects on carbohydrate digestive enzymes. Overall, the present study provided a detailed description of the phytochemical composition and biological effects of CLAE, opening new possibilities for the valorization of carob leaves for culinary and medicinal purposes.

## Figures and Tables

**Table 1:** *δ*(^1^H) and *δ*(^13^C) of the α-rhamnopyranoside moiety of myricetin-3-*O*-α-rhamnopyranoside.

	CH1″	CH2″	CH3″	CH4″	CH5″	CH_3_6″
*δ*(^1^H)	5.23	4.00	3.58	3.18	3.37	0.87
*δ*(^13^C)	103.82	71.93	72.29	73.18	72.72	19.38

**Table 2:** Identified analytes of the carob leaf acetone extract using UPLC-PDA-ESI/QToF.

	Component Name	Formula	Neutral Mass (Da)	Observed Mass (Da)	Mass Error (mDa)	Mass Response	Mass Fragments	UV λ-Max	Reference
1	Siliquapyranone	C_26_H_28_O_16_	596.13773	595.13050	0.0	2,683,020	169.01484 (100.00), 595.13052 (70.50), 443.11924 (59.54), 125.02533 (33.09)	275	[13,31]
2	Myricetin-3-*O*-α-l-rhamnopyranoside	C_21_H_20_O_12_	464.09548	463.08850	0.3	2,548,008	316.02282 (100.00), 317.02851 (45.59), 151.00440 (7.57)	353, 254	[13,30]
3	Myricetin-3-*O*-galactopyranoside	C_21_H_20_O_13_	480.09039	479.08290	−0.2	1,432,146	316.02261 (100.00), 317.02766 (32.19), 447.05651 (4.97), 151.00420 (4.35)	356, 258	[13,30]
4	Trigalloylglucose isomer 1	C_27_H_24_O_18_	636.09626	635.08980	0.8	1,165,685	169.01498 (100.00), 465.06770 (97.45), 313.05699 (80.55), 295.04649 (11.07)	278	[30,31,32]
5	Tetragalloylglucose isomer 1	C_34_H_28_O_22_	788.10722	787.10000	0.0	1,071,599	465.06758 (100.00), 169.01396 (77.28), 617.07876 (28.32), 313.05685 (25.76)	277	[30,31,32]
6	Gallic acid 4-*O*-glucoside	C_13_H_16_O_10_	332.07435	331.06710	0.0	996,079	169.01451 (100.00), 123.00836 (91.62)	267	[30,31,32]
7	Quercetin-3-*O*-alpha-l-rhamnopyranoside	C_21_H_20_O_11_	448.10056	447.09370	0.4	899,055	300.02766 (100.00), 151.00305 (10.01), 283.02804 (2.19)	347, 254	[1]
8	Digalloylglucose isomer 1	C_20_H_20_O_14_	484.08531	483.07830	0.2	879,802	169.01491 (100.00), 331.06726 (20.67), 313.05687 (17.75)	276	[2,3,4]
9	Digalloylglucose isomer 2	C_20_H_20_O_14_	484.08531	483.07820	0.2	574,116	169.01485 (100.00), 313.05660 (16.70), 331.06715 (16.29)	274	[2,3,4]
10	Galloylparasorboside	C_19_H_24_O_12_	444.12678	443.11950	0.0	322,674	169.01479 (100.00), 331.06722 (14.44), 313.05674 (11.38)	276	[13]
11	Gallic acid	C_7_H_6_O_5_	170.02152	169.01490	0.6	311,378	125.02507 (100), 151.00437 (8.23)	270	[30,31,32]
12	Myricetin Pentoside	C_20_H_18_O_12_	450.07983	449.07250	−0.1	220,353	316.02268 (100.00), 317.02781 (28.09), 151.00425 (5.15)	355, 265	[30,31,32]
13	Epigallocatechin 3-*O*-gallate	C_22_H_18_O_11_	458.08491	457.07740	−0.2	197,557	169.01485 (100.00), 125.02513 (45.07), 305.06664 (4.95)	276	[13,30,31,32]
14	Trigalloylglucose isomer 2	C_27_H_24_O_18_	636.09626	635.08940	0.4	134,091	465.06737 (100.00), 313.05660 (86.80), 483.07794 (43.64), 295.04602 (20.42)	276	[13,30,31,32]
15	Kaempferol/Luteolin Deohexoside	C_21_H_20_O_10_	432.10565	431.09830	−0.1	108,222	285.04055 (100.00), 284.03313 (58.55), 255.03055 (26.10)	345, 264	[30,32]
16	Digalloylglucose isomer 2	C_20_H_20_O_14_	484.08531	483.07790	−0.1	95,247	169.01468 (100.00), 313.05665 (33.31), 331.06693 (12.68)	274	[30,31,32]
17	Trigalloylglucose isomer 3	C_27_H_24_O_18_	636.09626	635.08940	0.4	80,731	465.06782 (100.00), 295.04619 (13.38)	275	[13,30,31,32]
18	Tetragalloylglucose isomer 2	C_34_H_28_O_22_	788.10722	787.09950	−0.4	59,869	617.07982 (100.00), 465.06764 (70.43), 313.05634 (37.56), 295.04610 (25.85)	278	[30,31]

**Table 3:** Minimum inhibitory concentration (MIC) and minimum bactericidal concentration (MBC) of carob leaf acetone extract against Gram-positive bacteria. MIC and MBC values are expressed as (μg mL^−1^).

Bacterium	MIC	MBC
*Bacillus cereus* ATCC 6089	150	250
*Listeria monocytogenes* ATCC 23074 (serotype 4b)	250	500
*Staphylococcus aureus* ATCC 6538	50	150

**Table 4:** Prediction of inhibitory effects of carob leaf acetone extract phytoconstituents with the employment of Antibac-Pred.

Compound	Confidence Score
Gallic acid 4-*O*-glucoside	0.7582
Galloyl-hydroxybenzoate-dihexoside	0.6588
Siliquapyranone (digalloyl-parasorboside)	0.6137
Myricetin-3-*O*-galactopyranoside	0.4524
Quercitrin (Quercetin-3-*O*-alpha-l-rhamnopyranoside)	0.4470
Myricitrin (Myricetin-3-*O*-α-l-rhamnopyranoside)	0.4447
Trigalloyl-hexose	0.4121
Digalloyl-hexose	0.3680
Tetragalloyl-hexose	0.269
Gallic acid	0.2158
Myricetin pentoside	0.1841
Epigallocatechin 3-*O*-gallate	0.0268

## Data Availability

The data presented in this study are available on request from the corresponding author.

## Supplementary Materials

The following supporting information can be downloaded at: https://www.mdpi.com/article/10.3390/molecules29225273/s1, Figure S1. Expanded OH-5 region of the ^1^H-^13^C HMBC spectrum of Figure 3; Figure S2. Mass Chromatograms of carob leaf acetone extract: (A) The major identified components, (B) Total Ion Count (TIC); PDA Chromatograms: (C) UV 254 nm (flavonoid 1st band), (D) UV 280 nm (phenolic acid band) (E) UV 360 nm (flavonoid 2nd band). Mass and PDA chromatograms are x-axis aligned; Figure S3. Structures of the major components of the acetone extract of *Ceratonia siliqua* L.
