# Supplementary material for: Phytochemical Analysis and Biological Evaluation of Carob Leaf (Ceratonia siliqua L.) Crude Extracts Using NMR and Mass Spectroscopic Techniques
Source: Molecules. 2024 Nov 7;29(22):5273. doi: 10.3390/molecules29225273 (PMC11596379; doi:10.3390/molecules29225273)
Supplement: Supplementary file 1 [file molecules-29-05273-s001.zip › molecules-3297298-supplementary.pdf]

SUPPLEMENTARY MATERIAL

Phytochemical Analysis and Biological Evaluation of Carob Leaf (*Ceratonia siliqua* L.) Crude Extracts Using NMR and Mass Spectroscopic Techniques

Themistoklis Venianakis[1], Nikolaos Parisis[1], Atalanti Christou[2], Vlasios Goulas[2*], Nikolaos Nikouladakis[2], George Botsaris[2], Tjaša Goričan[3], Simona Golič Grdadolnik[3] Andreas G. Tzakos[1], and Ioannis P. Gerothanassis[1]

[1] Section of Organic Chemistry and Biochemistry, Department of Chemistry, University of Ioannina, Ioannina, GR, 45110, Greece1; vethemis@gmail.com (TV); nparisis@uoi.gr (NP); atzakos@uoi.gr (AGT); igeroth@uoi.gr (IPG)
[2] Cyprus University of Technology, Department of Agricultural Sciences, Biotechnology and Food Science, 3603 Lemesos, Cyprus; atalanti.christou@cut.ac.cy (AC); vlasios.goulas@cut.ac.cy (VG); n.nikoloudakis@cut.ac.cy (NN); george.botsaris@cut.ac.cy (GB)
[3] National Institute of Chemistry, Laboratory for Molecular Structural Dynamics, Theory Department, Hajdrihova 19, SI-1000 Ljubljana, Slovenia; Tjasa.Gorican@ki.si (TG); simona.grdadolnik@ki.si (SGG)
* Correspondence: vlasios.goulas@cut.ac.cy; Tel.: 00357 2500141 (VG)

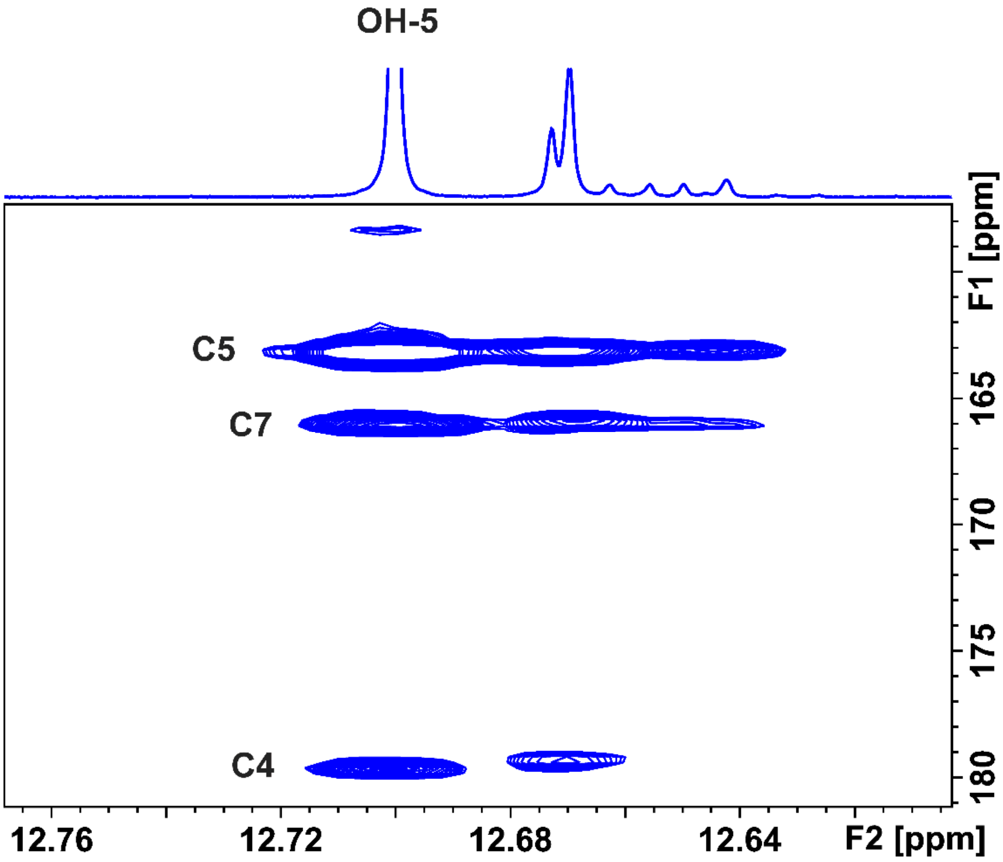

Figure S1. Expanded OH-5 region of the ^{1}H-^{13}C HMBC spectrum of Figure 3.

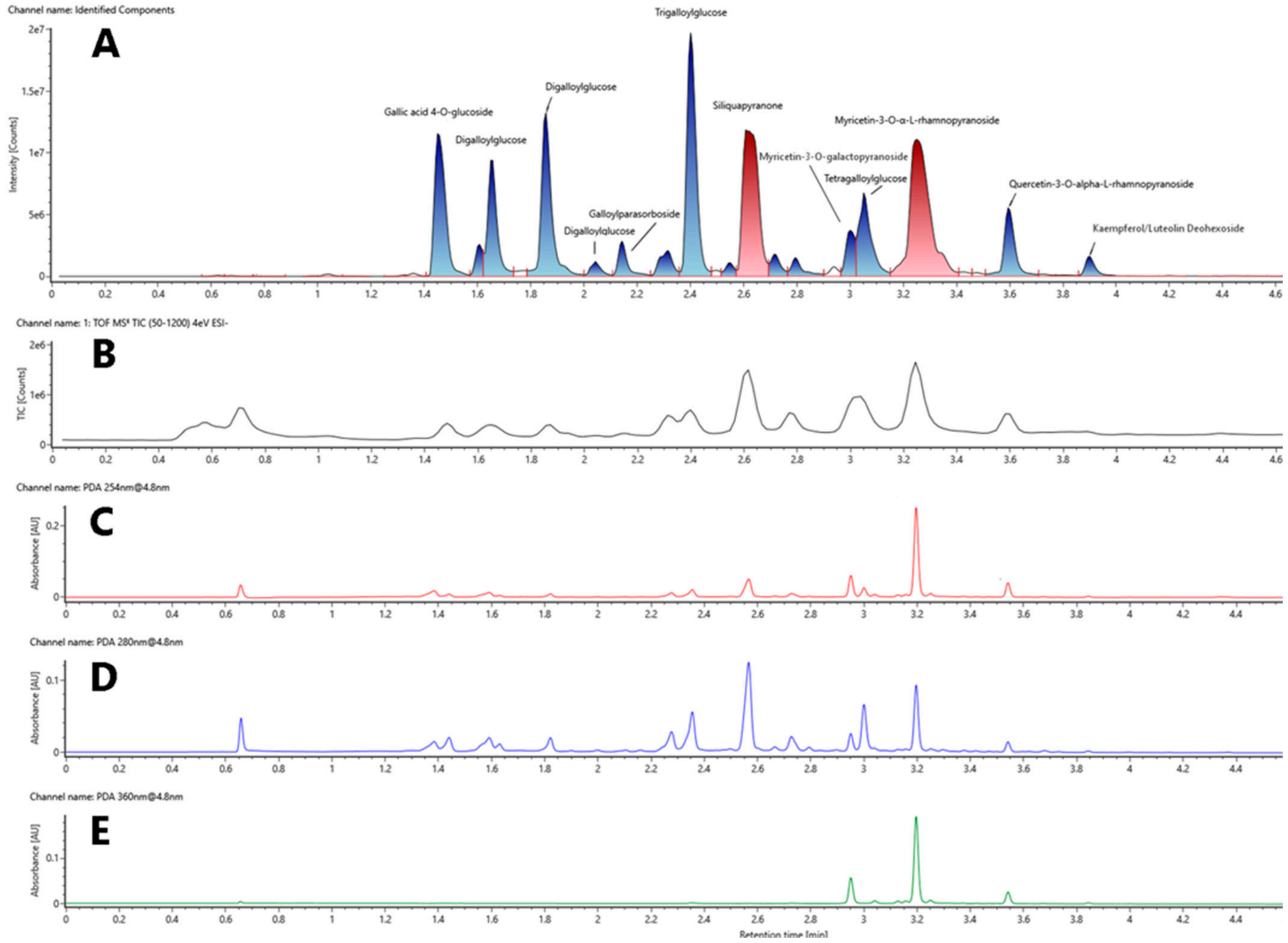

Figure S2. Mass Chromatograms of carob leaf acetone extract: (A) The major identified components, (B) Total Ion Count (TIC); PDA Chromatograms: (C) UV 254nm (flavonoid 1st band), (D) UV 280nm (phenolic acid band) (E) UV 360nm (flavonoid 2nd band). Mass and PDA chromatograms are x-axis aligned.

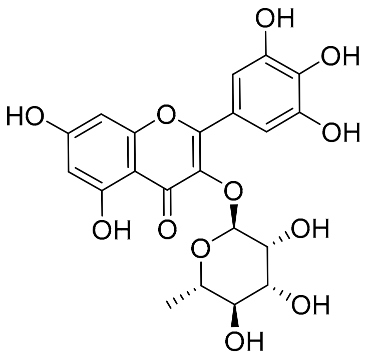

Myricitrin

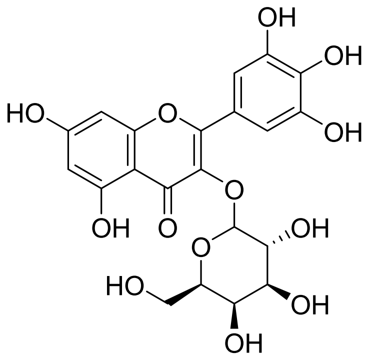

Myricetin-3-O-galactoside

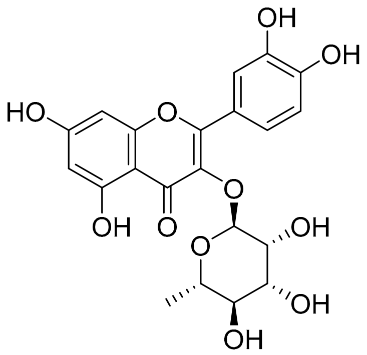

Quercitrin

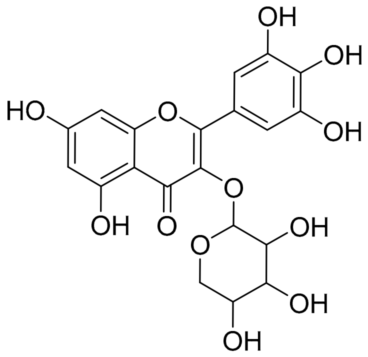

Myricetin-pentoside

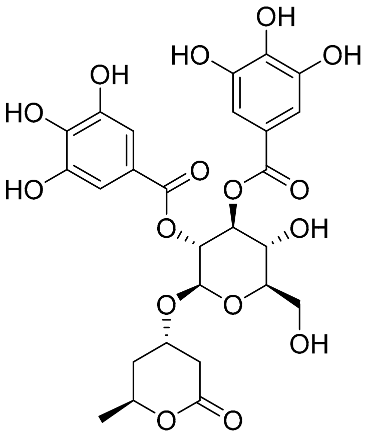

Siliquapyranone

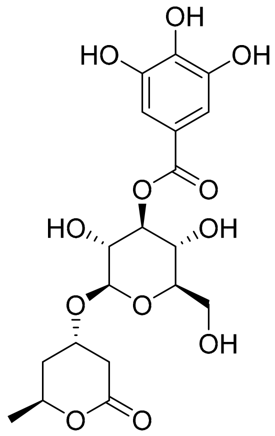

Galloyl Parasorboside

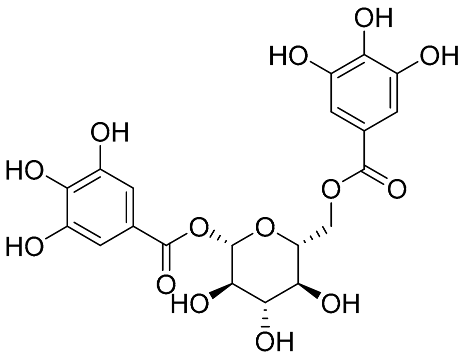

Digalloyl-glucose

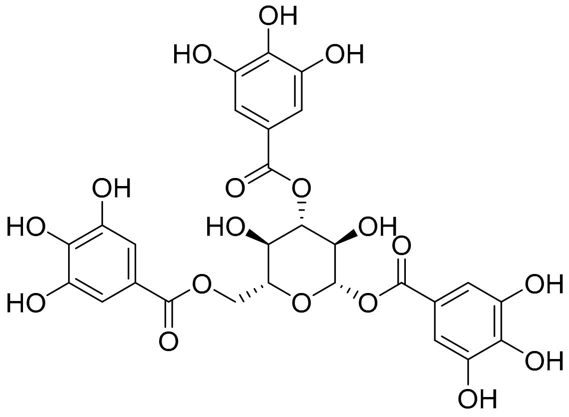

Trigalloyl-glucose

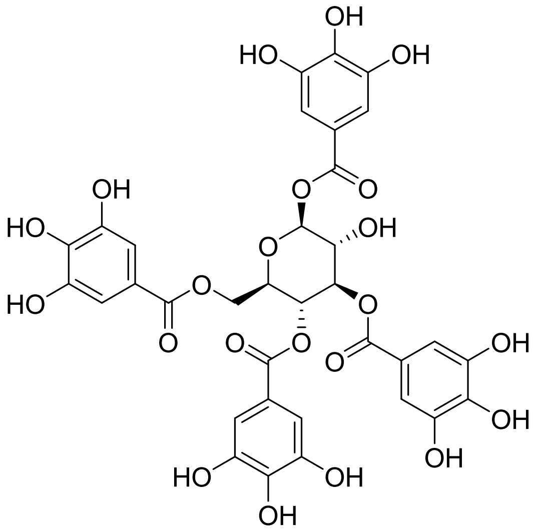

Tetragalloyl-glucose

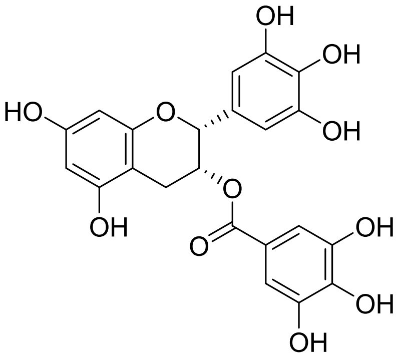

Epigallocatechin 3-O-gallate

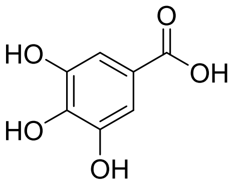

Gallic Acid

Figure S3. Structures of the major components of the acertone extract of *Ceratonia siliqua* L
